# Ethical Use and Impact of Participatory Approaches to Research in Post-Disaster Environments: An Australian Bushfire Case Study

**DOI:** 10.1155/2018/5621609

**Published:** 2018-06-11

**Authors:** L. Gibbs, K. Block, C. MacDougall, L. Harms, E. Baker, J. Richardson, G. Ireton, H. C. Gallagher, R. Bryant, D. Lusher, P. Pattison, J. Watson, J. Gillett, A. Pirrone, R. Molyneaux, S. Sexton-Bruce, D. Forbes

**Affiliations:** ^1^Jack Brockhoff Child Health and Wellbeing Program, Melbourne School of Population and Global Health, University of Melbourne, Parkville, Australia; ^2^Centre for Disaster Management and Public Safety, University of Melbourne, Parkville, Victoria, Australia; ^3^College of Medicine and Public Health, Flinders University, Adelaide, South Australia, Australia; ^4^Department of Social Work, University of Melbourne, Parkville, Victoria, Australia; ^5^Emergency Services, Australian Red Cross, Carlton, Victoria, Australia; ^6^Centre for Transformative Innovation, Swinburne University of Technology, Australia; ^7^Melbourne School of Psychological Sciences, University of Melbourne, Parkville, Victoria, Australia; ^8^School of Psychology, University of New South Wales, Sydney, New South Wales, Australia; ^9^Department of Education, University of Sydney, Sydney, New South Wales, Australia; ^10^North-East Primary Care Partnership, West Heidelberg, Victoria, Australia; ^11^Australian Rotary Health, Parramatta, New South Wales, Australia; ^12^Department of Health and Human Services, Melbourne, Victoria, Australia; ^13^Phoenix Australia-Centre for Posttraumatic Mental Health and Department of Psychiatry, University of Melbourne, Parkville, Victoria, Australia

## Abstract

This paper presents a case study of Beyond Bushfires, a large, multisite, mixed method study of the psychosocial impacts of major bushfires in Victoria, Australia. A participatory approach was employed throughout the study which was led by a team of academic investigators in partnership with service providers and government representatives and used on-site visits and multiple methods of communication with communities across the state to inform decision-making throughout the study. The ethics and impacts of conducting and adapting the approach within a post-disaster context will be discussed in reference to theories and models of participatory health research. The challenges of balancing local interests with state-wide implications will also be explored in the description of the methods of engagement and the study processes and outcomes. Beyond Bushfires demonstrates the feasibility of incorporating participatory methods in large, post-disaster research studies and achieving rigorous findings and multilevel impacts, while recognising the potential for some of the empowering aspects of the participatory experience to be reduced by the scaled-up approach.

## 1. Introduction

There are many different forms of participatory health research (PHR) but the shared principle is that “research is not done* on* people as passive subjects providing* data* but* with* them to provide relevant information for improving their lives. The entire research process is viewed as a partnership between stakeholders…” [1]. This aims to ensure that the people whose lives or work are the subject of the study have a central role in decision-making. Research activities in PHR create opportunities for cocreation of knowledge, and the different forms of expertise that each person brings to the process are valued [2]. There is also a shared commitment to achieve outcomes from the research that are of direct benefit to those involved.

There is growing evidence of the benefits of PHR in terms of recognition of different forms of expertise, increased relevance and uptake of outcomes, increased empowerment and/or self-efficacy, improved health status and health behaviours, and changes located in the social system [3–6]. However, there is limited evidence about the essential mechanisms to achieve change [7], an issue that a recent study by Lucero et al. begins to address in their evaluation of a conceptual framework for community based participatory research (CBPR) [8].

The uncertainty about mechanisms for change perhaps reflects the nature of PHR as an adaptive research approach incorporating many different forms of expertise, conducted in the complexity of real world situations. The strength of PHR is its contextual relevance [9] but this also offers the greatest challenge; the various forms of PHR defy attempts to define and confine them within structured guidelines [1, 10]. PHR has particular relevance for communities of disadvantage or potentially vulnerable groups because there is scope for their lived experiences to be recognised and to build their capacity and empower them in the process of shaping the research and outcomes [11]. This requires diligence in consideration of the ethics and impacts of research practice to ensure avoidance of the symbolic violence that arises if those involved are misunderstood or misrepresented [12]. Procedural ethics and review committees have been widely established in research institutions in many countries to guide the development and approval of study designs. However, an additional form of ethics in practice, referred to as “ethical reflexivity”, is proposed by Guilleman and Gillam to enable sensitive responses in situ to the complex issues that can emerge unexpectedly in health and social research [13]. Similarly, Banks et al. note the importance of relationship based approaches to ethics in participatory research [14]. The* Guide to Ethical Principles and Practice* has been produced and promoted by the International Collaboration for Participatory Health Research to provide helpful guidance in this field [9].

This paper presents a case study of Beyond Bushfires: Community Resilience and Recovery, a large, multisite post-bushfire research study. The purpose of this paper is to explore the multilevel processes involved for PHR and the importance of reflexive decision-making, particularly in sensitive contexts, and to highlight the subsequent impacts on stakeholders. The terms* impact* and* outcomes* can be used differently in evaluation. In this paper* outcomes* refers to specific changes that have occurred because of the program and* impact* refers to the effect of these changes on the stakeholders over time [1]. This case study is presented according to two core principles of the* Guide to Ethical Principles and Practice *which were found to have particular relevance, the principles of “equality and inclusion” and “making a difference”.

## 2. Background

In February 2009, intense bushfires raged across the regional areas of Victoria, Australia. The worst of the fires occurred on Saturday 7 February, which became known as “Black Saturday”. The impact on social and physical infrastructure was severe with 173 lives lost, over 2,000 homes destroyed, and entire communities devastated. The Vice-Chancellor of the University of Melbourne sent out a call to staff to see how the university could respond to this terrible event. In one response, a team of investigators with expertise relating to mental health, social connectedness, and wellbeing was formed and engaged in initial discussions with service providers and affected communities. It was agreed there was potential to make a considerable contribution to the evidence base to support the recovery of those affected and to inform ongoing disaster management policy and practice. The subsequent** Beyond Bushfires: Community Resilience and Recovery** mixed method study [15] was conducted from 2010 to 2016 and examined individual and community level resilience and recovery in the 3-5 years following the Black Saturday bushfires in 10 rural regions of Victoria, Australia, and involving 25 communities. A survey was circulated in 2012 to people living in the selected communities and those who had relocated, and 1,056 people participated either by phone interview or online. When the survey was repeated in 2014, 736 (78%) completed the survey again. Semistructured interviews were also conducted with 35 people aged from 4-66 years in 2013 and 2014. These interviews included a participant-guided mobile method where participants took the researchers around their homes, properties, and communities to show what was important to them [16]. There was no significant difference between participants in high, medium, and low impact communities in terms of sex, age, country of birth, or employment status.

## 3. Equality and Inclusion

The Guide to Ethical Principles and Practice [9] defines the role of equality and inclusion in PHR as follows:

  …*encouraging and enabling people from a range of backgrounds and identities (e.g., ethnicity, faith, class, education, gender, sexual orientation, (dis)ability, age) to lead, design and take part in the research, including a commitment to*:
  - seeking actively to include people whose voices are often ignored.
  - challenging discriminatory and oppressive attitudes and behaviours.
  - * ensuring information, venues and formats for meetings are accessible to all.* (p9)

The initial development of the Beyond Bushfires study proposal involved the formation of a community of scholars [17] in the immediate aftermath of the fires. The term ‘community of scholars' is used in this paper to refer to a group of people bringing different forms of expertise to the issue being researched, including academic, community, government and service provider representatives. The University of Melbourne allocated seed funding to make it possible to develop and commit to a program of research on the individual and community level impacts of the bushfires. It was led by Professor Elizabeth Waters, a public health academic who had expertise in and commitment to PHR, ensuring the organisational structures were in place to support participatory processes [18]. The community of scholars cogenerated the research topic and study design to enable preparation and submission of a detailed application for substantial Australian Research Council Linkage funding (LP100200164). Members of the community of scholars included a team of academic investigators with expertise in trauma, grief, public health, social networks, resilience, and community wellbeing. Organisational partners included the following: Victorian Department of Health, Australian Red Cross, Australian Rotary Health, Centrelink, Phoenix Australia: Centre for Posttraumatic Mental Health, and six Primary Care Partnerships. The government and organisational partners all had responsibility for disaster recovery services. The Primary Care Partnerships (PCPs) were regional collectives of health service providers and were the initial representatives for community perspective on bushfire recovery. The University of Melbourne as the academic lead also had overall responsibility for leading Beyond Bushfires, as is often the case in participatory studies involving substantial research funding and associated ethical processes [19]. The funding proposal was for a mixed method 5-year study examining the impact of the bushfires on mental health, wellbeing, and social connectedness in diverse communities across Victoria with different levels of bushfire impact, supported by participatory principles to guide decision-making and research processes throughout the study. Mixed and multimethod research designs can be usefully combined with participatory methods to address health research issues in a way that is meaningful to local communities and stakeholders [20].

**Figure 1:**
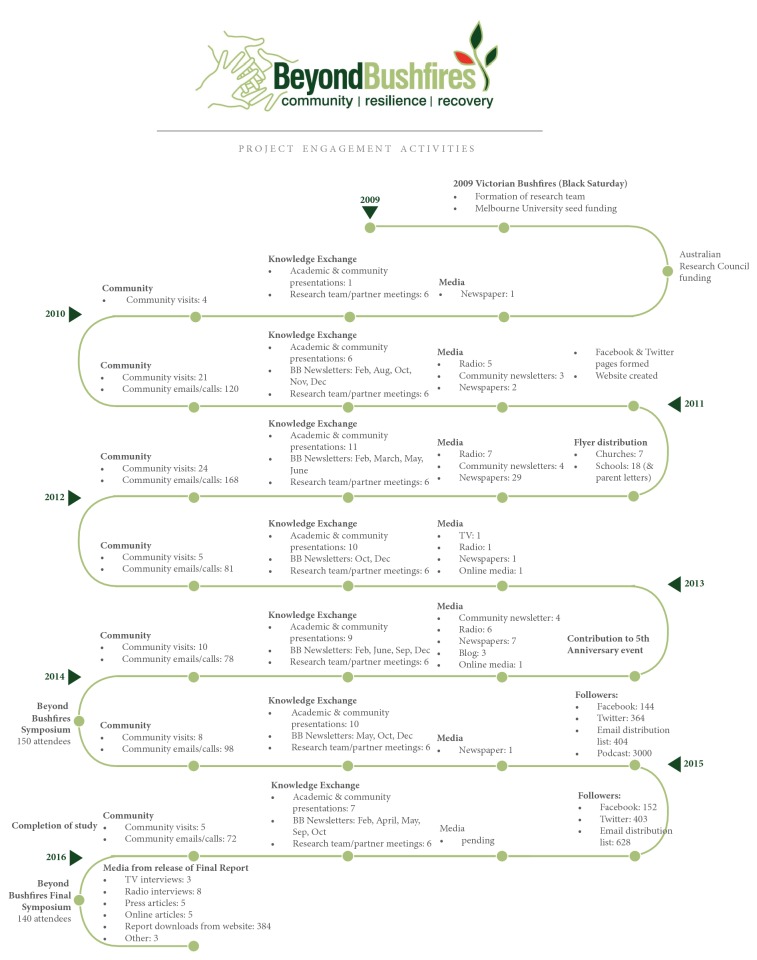


There were inevitable time delays in securing major research funding. Major competitive funding grants are not structured to respond quickly to sudden events such as disasters and tend to have low success rates. For this reason, it can be difficult and potentially unethical to invest fully in a participatory process if there are high levels of uncertainty and delays. The university's seed funding enabled us to commit the resources to cogenerate the competitive funding submission that was ultimately successful. When the PCPs were notified eight months later of the success in securing funding they were reluctant to continue to act as community representatives because they were no longer directly involved in the coordination of bushfire recovery services. This had been allocated to specially established bushfire recovery committees which varied in each location but were generally led by community members and had links to local government. In larger, more complex communities, the PCPs advised that there were many established and newly formed community groups and service provider committees that had different and sometimes competing roles in relation to bushfire recovery. This was the reality of the post-disaster environment and reflects the episodic nature of leadership that shifts in response to changed processes and context [21]. So we asked our PCP partners to direct us to all relevant contacts in their local communities who were involved in the bushfire recovery process or were connected to community information sharing networks. The relevant contacts varied considerably so in each community we asked them “who should we be speaking to?” (Yes, we know this is not good grammar!) and following these instructions we embarked on a series of formal and informal community visits that included meeting with community leaders, walking around town with a local host, and attending meetings of different committees, community groups, and networks to listen to their local issues and talk to them about the research (see Figure 1). In those initial encounters we presented the research concept and asked if they would accept our invitation to engage in a research partnership with their community so that we could work together to develop and conduct the research. This took time and we developed a better sense of the interests and needs of the communities as we went. All but one of the communities responded very positively to this approach. The community that declined was the first one we spoke to. They explained that they felt the research was important and they understood this approach would require an investment from them but in the post-disaster circumstances they did not have the energy or capacity to accept. We always wondered whether they would have accepted if they had been one of the last ones we approached almost a year later, when things were slightly less chaotic. We did not reapproach them out of respect for their considered decision to decline the invitation.

Initially we anticipated that we would invite community members to be part of our bimonthly research investigator and partner meetings because that was where we anticipated most of our decisions would be made but through the early process of community visits and attending community meetings relating to the bushfire recovery, it increasingly became clear that there were many competing voices and tensions over people acting as unelected spokespersons. The community visits were conducted in pairs providing the opportunity to debrief and reflect on the journey back. This ethical reflexivity extended into our combined investigator and partner meetings where the post-disaster political environment, including a Victorian Bushfires Royal Commission conducted from 2009 to 2010 to investigate the causes and responses to the bushfires, was also taken into account in our decision-making [13, 22]. The process of reflexivity in our discussions included consideration of how the research processes and the broader post-disaster environment could have an unintended influence on community members' experience of the research and the research outcomes. These challenges in determining who should be involved and how should public participation be achieved are common in participatory approaches [23, 24]. As a result, we decided to engage in an ongoing process of community visits and communications to enable us to hear the multiple voices and perspectives rather than rely on a small group of community representatives. Taylor et al. [25] in their application of a Scottish participation framework to an Australian rural multisite participatory study identified “having the right people involved” as an essential factor to success (pg. e102). In the post-disaster context, it was unlikely this could be achieved with a small number of people so we endeavoured to make as many community connections as possible. This was achieved initially through an extensive process of engagement over 12 months contacting and visiting selected communities to discuss the proposed study, to check if it seemed the right approach and if there was local support for participation in the study, and to discuss local contextual considerations and sensitivities.

Comments and suggestions were made by each of the participating communities throughout the study which contributed to progressive adjustments to the study design and measures, enacting Freire's praxis in the development and conduct of the study [26]. Our efforts to operate ethically and sensitively in a highly politicised and emotive environment may have inadvertently undermined community members' right to self-determination in the study, i.e., by visiting multiple sites and collecting multiple perspectives, we reduced the opportunities for a single group of community members to take responsibility for the decision-making and thereby may have limited the capacity for joint critical reflexivity and collective action [1]. However, community members demonstrated that they valued this neutrality by their willingness to include us in neighbourhood and committee meetings and to direct us to speak with other groups with competing views. Sometimes we received long phone calls and emails from community members who felt the research was important and wanted to make a contribution but had decided not to attend community meetings anymore because of previous experiences of hostility in that context. The high level of trust in the researchers was particularly notable given reports of earlier negative experiences with insensitive research practices. Another university had sent a questionnaire and consent form directly to residents in affected areas soon after the fires. Many service providers told us that the community members had been extremely distressed to open an unsolicited letter and be confronted by questions about their bushfire exposure. Instead of this nonparticipatory approach, we used our community visits to guide us. We shared information about our study through local networks before we even began a recruitment process, and we established a sensitive research protocol to minimise any potentially distressing impact of our research processes. The protocol included the community based participatory approach; obtaining informed consent before presenting the survey material about the bushfires; referral material for support services; a participant-guided approach for qualitative interviews; checks and progressive consent embedded within the surveys to alert people to upcoming questions about disaster exposure; closed questions about trauma exposure; and mental health supports [27].

The post-disaster period is generally marked by initial bonding from the shared experience of the incident, followed often by a fracturing of personal and community relationships [28]. Respecting diverse perspectives proved to be an important means of navigating through this constantly shifting social context [14]. It also allowed us to see the common issues arising at different stages of the recovery processes as well as the differences arising from personal circumstances and contextual influences. For example, in the early stages of recovery many community members who were affected by the bushfires were reluctant to access support services and resources in deference to those who were perceived to have lost the most. However, over the next couple of years tensions arose as different forms of support were disseminated and there was a perception that some people who had not really been adversely affected were benefiting unfairly from donated goods and recovery grants. The involvement of partner agencies provided important insights at all stages, including Red Cross advice on a humanitarian response; Phoenix Australia advice on care in a post trauma context; and government advice on current service delivery and uptake.

We also had to manage the differing perspectives and approaches of the team of academic investigators. An initial academic workshop was held so that each of the investigators could present their field of expertise, explain what they brought to the process, what were the required standards of rigour for their methods, and the research gaps they were interested in addressing. This proved to be instrumental in embarking on a transdisciplinary approach throughout. For example, it emerged that our epidemiologists needed to use random sampling for the survey, while our social network analysts needed snowball sampling. As a result we engaged in saturation sampling within selected communities; this satisfied both paradigms.

The insights we gleaned from our community visits, phone calls, emails and social media were always brought back to include in the decision-making processes with the academic and organisational partners. This influenced all aspects of the research including the study name, the study design, the research questions, the terminology (e.g., do not use the word “victim”), the recruitment zones, the questions in the survey, recruitment and information sharing methods, the focus of analyses, and dissemination of findings. For example, we consulted with community members about the recruitment zones for their community and discovered the area maps were not a useful guide for inclusion/exclusion. We expanded the recruitment zones to neighbouring towns when told the map boundaries were meaningless and the residents in the neighbouring areas were so closely connected it would be offensive to exclude them. Information about the study and recruitment processes was disseminated differently in each community, utilising local information networks. Repeatedly hearing emotional discussions about decisions on whether to stay living in the disaster affected community or to move away guided us in our sampling to include people who had relocated and ensured we also addressed this issue in our research questions and analyses [29]. The extensive and ongoing efforts to engage with different stakeholder groups across the state were only feasible because we had substantial research funding for an extended time. It was similar to other participatory studies in terms of consulting with citizens about research topics, priorities, and methods [30, 31] but the process did result in final decisions being made by the researchers and organisational partners, which is counter to the ideals of PHR [32–34]. Consistent with the findings of a systematic review of participatory research studies, participation was lowest regarding financial responsibility for the research funds [35].

In the early stages of Beyond Bushfires we asked community members if it should be presented as a University of Melbourne study or would it be better to be led by Australian Red Cross or community agencies to ensure sensitivity to local experiences. We were repeatedly told that the Black Saturday fires were such a major event that it made sense that a respected academic institution should lead the research process to ensure that we maximise the learnings. Perceived relevance of the findings was reinforced by informal feedback received throughout the study and indicated by over 3,000 downloads by community members of a podcast of one community seminar providing an overview of the study findings. Social media tweets from the final symposium reached 3,388 stakeholders. The different communication channels also provided opportunities for negative feedback which were valued in guiding the processes. For example, at one symposium we presented findings that after 3 years the majority of respondents were recovering, but a significant minority (approximately twice the levels evident in the general population) were showing signs of poor mental health [36]. Audience members were very unhappy with this finding and suggested that in their experience the levels of poor mental health were far greater. The resultant discussion changed the way we presented the research findings, taking care to note that the levels being presented referred to signs of diagnosable mental health conditions which may require clinical care, were likely to be under-reported and did not include the many people likely to be experiencing lower levels of impact on mental health and wellbeing. It is possible that community members and other stakeholders who were not comfortable with our research approach chose not to engage with the study or related events at all. Indeed, the intention of our initial awareness raising activities (see Figure 1) was to give them the opportunity to make a decision about participation before they were approached.

Repeated turnover of representatives of stakeholder groups, particularly in government departments, did make it difficult to maintain partnerships over the six years of the study. This was offset by the ongoing commitment by the research team and the partner organisations to the collaborative process so that handovers were arranged and briefings held to enable new contacts to come on board quickly. Internal presentations were provided when needed for all government and organisational partner agencies to share and discuss research findings with their staff/membership, to provide an opportunity for them to contribute insights into the data analysis and dissemination strategies, and to guide their own service delivery.

## 4. Making a Difference

The Guide to Ethical Principles and Practice [9] suggests that “making a difference” in PHR refers to the following:

  …*promoting research that creates positive change for communities of place, interest or identity*… (p10)

One of the primary goals of PHR is to generate positive change for those involved [9, 11]. In a large multisite study it is not possible for all stakeholders to have the direct, empowering experience of cogenerating the research process and outcomes that is a typical feature of PHR [1, 2]. Instead, we used and adapted PHR principles to reflect community issues and achieve outcomes that resonated for community members who were not directly involved, in an effort to conduct research that was experienced as positive, respectful, and relevant. In this sense, the intent was* substantive*, as described by Blackstock et al. [37] in the sense that “encouraging multiple perspectives improves understanding of the issues, and therefore the selection of appropriate solutions” (p727). The approach used was consistent with the model for community based participatory research (CBPR) with significant contributions from service providers and policy makers in addition to community members [1, 38–40]. There are other examples of large multisite, sometimes international studies using participatory methods to address health issues [41, 42].

Predicting and capturing the long-term benefits of participating in PHR studies are challenging, as shown by a review of 60 community based participatory health research studies by Viswanathan and colleagues [43]. For Beyond Bushfires, the impact of the participatory experience on stakeholders was not systematically assessed other than monitoring and responding to feedback received on the study processes, and participant reported experiences of interviews and questionnaires. Analysis of the questionnaire responses showed that the vast majority of participants were glad they participated even if they felt distress while reflecting on the questions [27]. Many people commented in their interviews about how it aided their own reflection about their experiences or that they felt it made a contribution for others:

 
*Well that's what it's all about isn't it. That's what we've been banging on about, information sharing to save somebody else or to influence something*.

One of the community leaders who had been very positive about the study in her own presentations was asked to provide a quote for a University of Melbourne annual report. She reported the following:

The Beyond Bushfires study provides a unique window into recovery from an individual and community perspective. It has helped us to understand what we are seeing in ourselves and others, to know what is to be expected, and conversely, what is not. Most importantly, it has provided a safe, supportive environment for us to explore the lived experience of bushfire recovery…The Beyond Bushfires project and linked research has helped to make sense of the way recovery evolves over time. It has added significantly to our understanding and validated our perceptions and insights…Participating in the research was both empowering and cathartic.

Without agreed measures of impact in terms of the effect of the research processes and outcomes on the stakeholders involved, it is impossible to demonstrate the benefits of the participatory processes with any strength of evidence. Inclusion of a cogenerated framework for evaluating the impact of the participatory elements of Beyond Bushfires would have strengthened the study design [1, 37].

**Figure 2:**
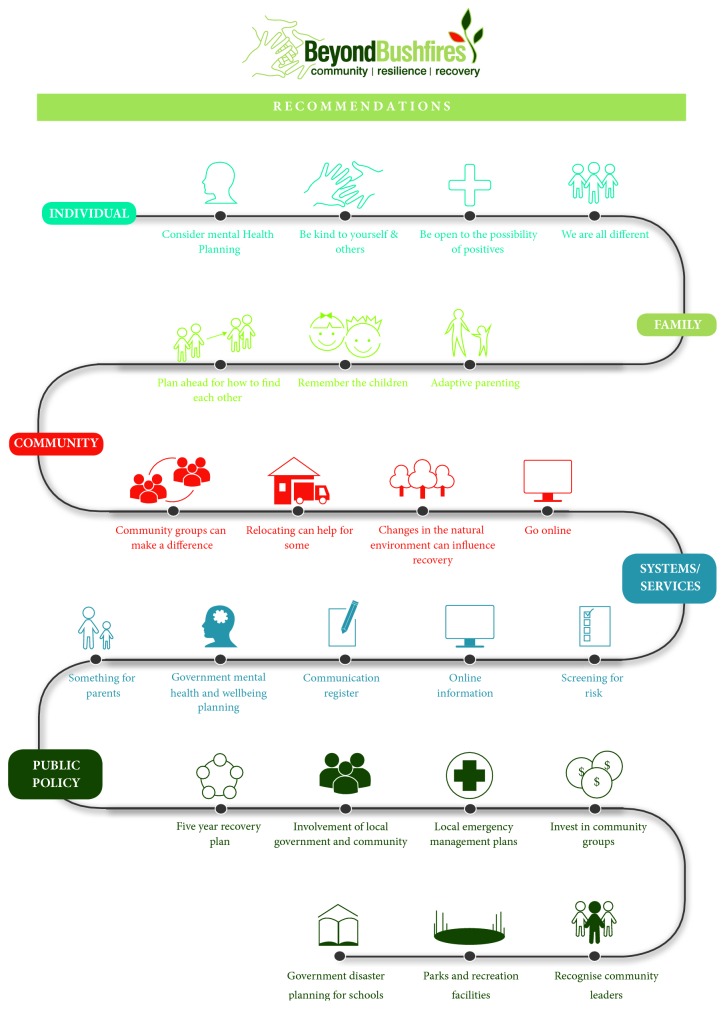


The participatory processes in the Beyond Bushfires study did make it possible to identify evidence gaps that needed to be addressed to guide post-disaster decision-making at individual, community, service provider, and government level. This was reflected in a subsequent mapping of key Beyond Bushfires research findings and recommendations using a socioecological framework to provide clear guidance to stakeholders about opportunities to promote positive impacts at individual, family, community, systems/services, and public policy levels (Figure 2). Additional information about these findings and recommendations can be found in the final report [44]. Partner organisation, Australian Red Cross, identified one of their evidence gaps in relation to separation. They are responsible for providing reunification services in emergency events. They requested inclusion of survey questions about the immediate separation of family members during and after the bushfires. This enabled analysis of duration and impact of separation [45], and methods of recontact, which has been used by Red Cross to guide their review of their reunification services, Register Find Reunite [46], and will thus contribute to sustainable change promoting health and wellbeing [1].

This cogeneration of knowledge has resulted in increased relevance and application of the research and researcher understanding of the complexities of disaster recovery and contextual influences on individual and community impact. For example, the Beyond Bushfires research showed that involvement in community groups is a strong protective factor for individual mental health impacts [44], consistent with other disaster related research which has shown the importance of social capital [47, 48]. However, community visits showed that decisions about rebuilding of community facilities sometimes promoted involvement and sometimes deterred it, if it forced different groups who did not get along to now share the same venue.

The involvement of government, service provider and community organisation partners also contributed to research translation into policy and practice. The final Beyond Bushfires symposium, held in October 2016, provided an opportunity for 140 stakeholders (community 30%, academics 30%, and government and service providers 40%) to discuss the findings and potential multilevel recommendations, and to consider next steps. The aim was to create a final communicative space in the research process to encourage shared learning and outputs [49]. It was encouraging to find that issues raised by the panel speakers and the audience, such as the importance of building knowledge about how to strengthen the disaster resilience of school communities, are already being addressed in new collaborative research projects that have emerged from Beyond Bushfires and involve the existing study partners and the Department of Education and Training. This is an example of the unexpected benefits that can emerge from a participatory process and contribute to ongoing changes promoting better health [1]. Additional community seminars were organised by local partners and embedded in local events to share and discuss the findings. A plain language report was also produced and distributed widely through the different stakeholder networks to ensure the accessibility of the findings and recommendations [44]. These different outputs are consistent with one of the goals of participatory research, to allow the different contributors to determine how best to report on and share the findings [50].

Each organisational partner engaged in a different way and had different outcomes, according to their core operations and interests [43]. The academic partners increased their expertise in disaster recovery research, understanding of community, policy and practice issues, and developed an ongoing research program and collaborations with the study partners. Having Australian Red Cross involved, as a national organisation with a clear organisational goal in supporting people affected by disasters, enabled the knowledge ascertained through the research to be incorporated into their own practice, including the Register Find Reunite service, as well as contributing to their advocacy for sound policy development in the emergency management sector. The Australian Government Department of Human Services funded a PhD scholarship within the study which was undertaken by a senior social worker employed by their Centrelink Service responsible for welfare payments. The Victorian Department of Health and Human Services used the partnership to inform their policy and practice in relation to disaster recovery psychosocial services, including consideration of disaster impact in their 10-year Mental Health Plan for the State of Victoria. Australian Rotary Health was able to share the research findings with service clubs throughout Australia. Phoenix Australia: Centre for Posttraumatic Mental Health are using the research findings to inform their trauma counselling training courses and to progress their research program. The Primary Care Partnerships use the research to inform local service delivery. Community members are using the research findings to inform their personal decisions about recovery and to make sense of their individual and community level experiences. A central goal of PHR is to “give primacy to the local context” to ensure local relevance and impacts [1]. In Beyond Bushfires it was possible to provide differentiated findings relating to level of bushfire affectedness in different areas and to incorporate influencing factors arising from local contexts. This provided outcomes which had local relevance despite the size and spread of the study.

The publication to date of 21 Beyond Bushfires academic articles in high ranking journals (http://www.beyondbushfires.org.au/) demonstrates that academic rigour and contribution to evidence was not compromised by this PHR approach and the subsequent stakeholder influence on study design and research processes. This is supported by the Viswanathan et al. review which showed the capacity of community based participatory research (CBPR) studies to implement high quality research methods, contributing to positive health outcomes [43].

**Figure 3:**
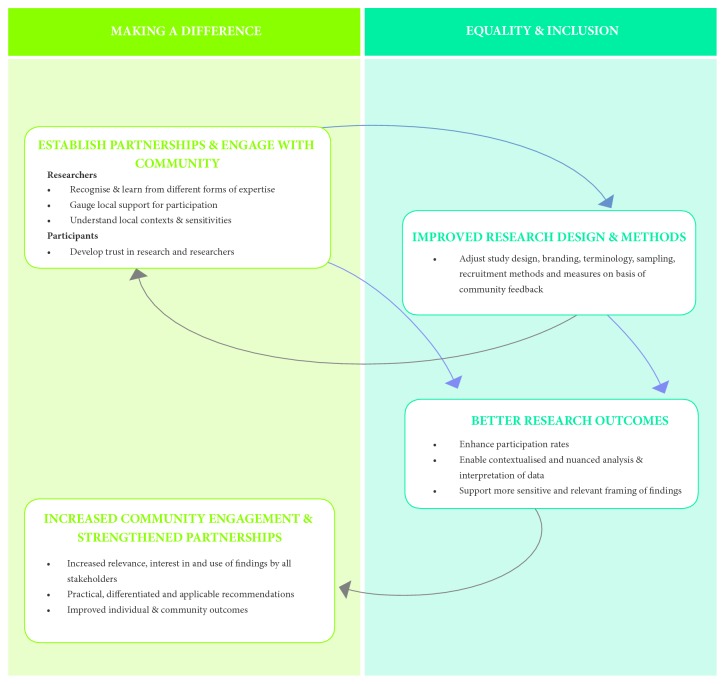
Outcomes of PHR: a virtuous cycle.

In summary, the multilevel outcomes of the Beyond Bushfires study achieved in the context of ethical and reflexive processes demonstrate a virtuous cycle between the “making a difference” and “equality and inclusion” aspects of the participatory approach (Figure 3) [9].

## 5. Conclusion

The inclusive research activities and the reflexive processes engaged in by the Beyond Bushfires community of scholars reflected a commitment to ethical and sensitive approaches and meaningful outcomes in a post-disaster environment. The participatory approach was adapted at community level to allow a scaled-up approach while still accommodating local tensions and contextual differences. It became clear in the early stages of the study that making many community connections over multiple sites was more likely to capture the multiple voices and perspectives rather than working closely with a small group of unelected community spokespersons. This made it possible to reflect the wide ranging experiences for the large number of communities and stakeholder groups that were involved and to achieve wider scale outcomes through the government and agency partners, but is likely to have reduced the potential for individual empowerment that would typically be ascribed to a participatory process. The time and funding that was available for this study made it possible to invest in the participatory process in a way that is not always possible in research studies. Ongoing stakeholder engagement and multilevel research outcomes indicate the participatory processes were positive and worthwhile. This demonstrates the value of the investment in relationships, site visits, recognition of different forms of expertise, and an adaptive approach to the development and implementation of the study design and research processes. Other factors which contributed to success were the use of different strategies customised to local contexts to engage with community members and share information; valuing multiple perspectives; and incorporating sensitivity protocols. While the impacts of the PHR processes in this study were not systematically monitored or measured, this descriptive account is intended to contribute insights into ethical applications of PHR in a large, multisite, mixed methods, post-disaster study.
